# Cutaneous and Subcutaneous Metastases From Atypical Laryngeal Carcinoids: Case Report and Review of the Literature

**DOI:** 10.1097/MD.0000000000002796

**Published:** 2016-02-18

**Authors:** Kui-Rong Wang, Yuan-Jing Jia, Shui-Hong Zhou, Qin-Ying Wang, Yang-Yang Bao, Zhi-Ying Feng, Hong-Tian Yao, Jun Fan

**Affiliations:** From the Department of Anaesthesia (K-RW, Z-YF); Department of Otolaryngology (Y-JJ, S-HZ, Q-YW, Y-YB); Department of Pathology (H-TY); and State Key Laboratory for Diagnosis and Treatment of Infectious Diseases, The First Affiliated Hospital, College of Medicine, Zhejiang University, Zhejiang Province, China (JF).

## Abstract

The incidence of cutaneous and subcutaneous metastases from atypical laryngeal carcinoids is approximately 20%. However, the pathogenesis and natural history of, and prognostic factors for, the condition remain poorly understood. We reported a 54-year-old female presented with cutaneous and subcutaneous metastases from atypical laryngeal carcinoid. Laryngoscopy revealed a 0.5 × 1.5-cm reddish mass on the laryngeal surface of the epiglottis. Under general anesthesia, a biopsy sample was obtained via suspension laryngoscopy. Routine pathology revealed atypical laryngeal carcinoid. Immunohistochemical staining of the sections of primary tumor was positive for cytokeratin, chromogranin A, synaptophysin, hypoxia-inducible factor-1α, P53, and CD56. GLUT-1, p-Akt, and PI3K were negative. The Ki-67 index was 15%. Supraglottic laryngectomy and selective right-neck dissection were performed. After 6 months, the patient complained of pain in the right wall of the chest; multiple cutaneous and subcutaneous nodules were evident at that site and in the abdomen. An abdominal nodule was biopsied and pathology revealed that the atypical metastatic carcinoid had metastasized to both cutaneous and subcutaneous areas of the abdomen. Chemotherapy was then prescribed. Currently, the intrathecal drug delivery system remains in place. No local recurrence has been detected. Furthermore, we systematically reviewed clinical manifestations of the disease, pathogenesis, prognostic factors, and treatment. The metastasis rate (cutaneous and subcutaneous) was approximately 12.2%. Thirty patients (62.5%) with cutaneous and subcutaneous metastases exhibited contemporaneous lymph node invasion. The 3-, 5-, and 10-year survival rates were 44.0%, 22.0%, and 13.0%, respectively.

The prognosis of patients with atypical laryngeal carcinoids was poor. Relevant prognostic factors included the level of p53, human papilloma virus status, certain hypoxic markers, and distant metastasis. No optimal treatment for such metastases has yet been defined.

## INTRODUCTION

Laryngeal neuroendocrine carcinomas (NECs) are rare, constituting <1% of all tumors of the larynx. Four histological subtypes are distinguished based on the extent of differentiation and cell size. Well and moderately differentiated NECs are termed typical and atypical carcinoids, respectively. Poorly differentiated NCLs are divided into small- and large-cell NECs.^1,2^ The most frequent laryngeal NEC is the atypical carcinoid, followed by small-cell NEC, carcinoid tumor, and large-cell NEC.^3^

The prognosis of laryngeal NEC varies by histopathological type.^2^ A meta-analysis of 436 patients with laryngeal NECs found that the 5-year disease-specific survival was 100% for patients with typical carcinoids, 53% for those with atypical carcinoids, and 19% for those with small-cell carcinomas. Prognostic factors included distant metastasis. An atypical carcinoid of the larynx is a more aggressive type of NEC, often associated with multiple distant metastases.^4,5^ Metastatic sites include the cervical and distant lymph nodes, lung, bones, skin, subcutaneous tissues, mediastinum, liver, heart, pancreas, diaphragm, peritoneum, gastrointestinal tract, prostate, breast, brain, dura mater, pleura, testicles, and muscles.^4^ Lymph node metastases are the most common (40%), followed by skin and subcutaneous metastases (20%), and metastases at other sites (40%).^6–8^ The prognoses of patients with atypical laryngeal carcinoids are relatively poor; the 5-year survival rate is approximately 50%. Death is usually caused by metastatic disease rather than local recurrence.^4^ Although the incidence of cutaneous and subcutaneous metastases from atypical laryngeal carcinoids is approximately 20%, few systematic analyses of clinical manifestations or treatment of such metastases have been reported. The precise means by which distant metastasis and local recurrence develop remain unclear, as do relevant prognostic factors.

To date, the only effective treatment appears to be surgery. The disease is refractory to chemotherapy, and any role for radiotherapy is controversial.^9^ Thus, new treatments are required to improve long-term survival. Targeted therapies have recently been used to treat other cancers,^10,11^ including NECs of other sites. Targets include vascular endothelial growth factor, platelet-derived growth factor, and the mammalian target of rapamycin; such treatments have improved the progression-free survival times of patients with pancreatic NEC, pulmonary large-cell-type NEC, and prostate NEC.^12–14^ However, no report on targeted therapy of laryngeal NEC has yet been described.

Our previous study^15^ and additional work^16^ have shown that positron emission tomography/computed tomography (PET/CT) detected high-level uptake of [18F]-fluoro-2-deoxy-d-glucose (FDG) by laryngeal NECs, as is also true of other head-and-neck cancers.^17–20^ Many studies have found that FDG uptake is associated with overexpression of glucose transporter-1 (GLUT-1),^20–23^ which is associated with metastasis and poor prognosis of many human cancers.^22,24,25^ NECs also express high levels of GLUT-1^26–28^ with certain biological consequences.^26^ We previously found that targeted inhibition of GLUT-1 decreased glucose uptake by, and inhibited proliferation of, Hep-2 cells,^29^ and enhanced the radiosensitivity of laryngeal carcinoma Hep-2 cells.^30^ Thus, we proposed that GLUT-1 targeting may be useful for the treatment of laryngeal NECs.

Here, we report a patient exhibiting cutaneous and subcutaneous metastases from an atypical laryngeal carcinoid and review clinical manifestations, possible pathogenesis, prognostic factors, and treatments. We measured the levels of GLUT-1 mRNA and protein, and screened for human papilloma virus (HPV), cytomegalovirus (CMV), and Epstein–Barr virus (EBV).

## CASE REPORT

### Presenting Concerns

A 54-year-old female presented with a sore throat and radiating pain in the right ear lasting over 1 year in duration. She denied hoarseness, respiratory distress, dysphagia, and fever. Her past medical history included hypertension during the 3 years prior, blood pressure was controlled by oral irbesartan (10 mg, per day).

### Clinical Findings

**FIGURE 1:**
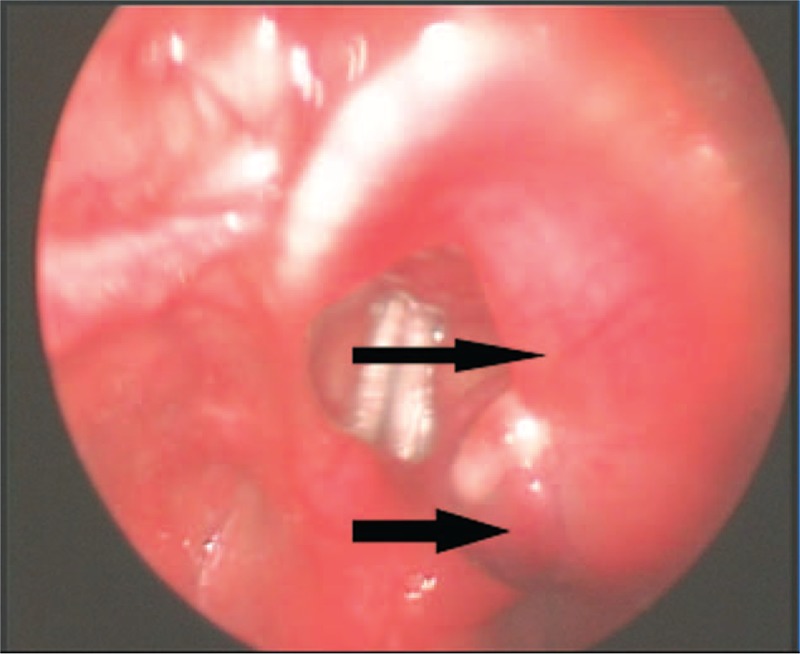
Laryngoscopy revealed a 0.5 × 1.5-cm reddish mass on the laryngeal surface of the epiglottis.

**FIGURE 2:**
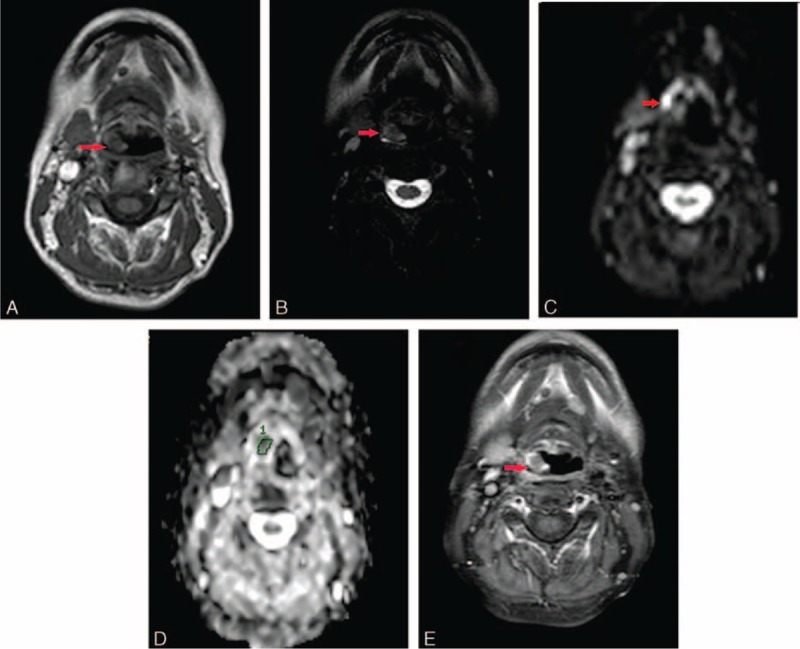
MRI revealed a 0.8 × 1.3-cm lesion in the epiglottis. T1-weighted imaging was isointense (A), but T2-weighted imaging was hyperintense (B). diffusion-weighted imaging exhibited high signal intensity (C). The apparant diffusion coefficient value was 0.741 × 10^−3^ mm^2^/s (D). The lesion exhibited heterogeneous enhancement (E). MRI = magnetic resonance imaging.

Laryngoscopy revealed a 0.5 × 1.5-cm reddish mass on the laryngeal surface of the epiglottis (Figure 1). Movements of both vocal cords were normal. Magnetic resonance imaging (MRI) revealed a 0.8 × 1.3-cm lesion in the epiglottis. T1-weighted imaging was isointense; T2-weighted imaging hyperintense; and diffusion-weighted imaging of high signal intensity. The lesion underwent heterogeneous enhancement following administration of gadolinium-DTPA (Figure 2). However, the preoperative PET/CT was not done.

The institutional review board (IRB no.2015427) of The First Affiliated Hospital, College of Medicine, Zhejiang University (Hangzhou City, China) approved the present study. Written informed consent was obtained from the patient before inclusion.

### Therapeutic Focus and Assessment

**FIGURE 3:**
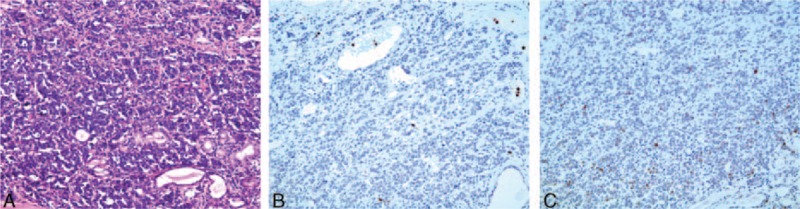
Pathology revealed that the tumor cells were uniform in size, markedly heteromorphic with eosinophilic cytoplasm, and formed nests and cords containing prominent blood sinuses (A) (HE × 200). Immunohistochemical staining was positive for hypoxia-inducible factor-1α (HIF-1α) (B) and p53 (C) (EliVision, ×200).

**FIGURE 4:**
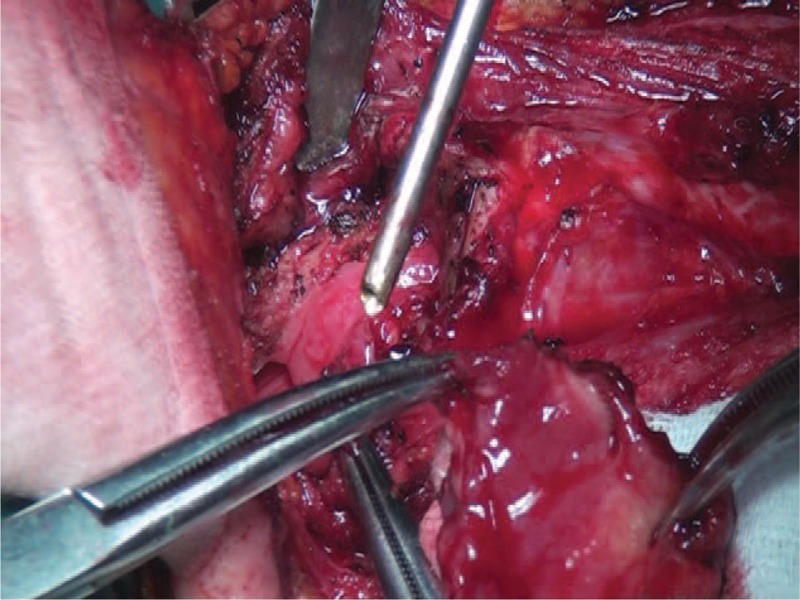
Surgical section revealed a reddish lesion in the laryngeal surface of the epiglottis.

The process of the patient received was listed in Table 1. Under general anesthesia, a biopsy sample was obtained via suspension laryngoscopy. Analysis of the frozen section suggested that only a small-cell tumor was present; therefore, it was necessary to immunohistochemically differentiate a myoepithelial tumor from an atypical carcinoma. Routine pathology revealed that the tumor cells were uniform in size, exhibited marked heteromorphy, had an eosinophilic cytoplasm, and were arranged into nests and cords containing prominent blood sinuses. Immunohistochemical staining of the sections of primary tumor was positive for cytokeratin, chromogranin A, synaptophysin, hypoxia-inducible factor-1α (HIF-1α), P53, and CD56. GLUT-1, p-Akt, and PI3K were negative. The Ki-67 index was 15% (Figure 3). These histopathological features confirmed the presence of an atypical carcinoid. On May 7th 2014, supraglottic laryngectomy and selective right-neck dissection were performed under general anesthesia (Figure 4). Postoperatively, the histopathological features of paraffin sections were confirmed to be those of an atypical carcinoid; the right cervical lymph nodes and the resection margin were negative. The pathological diagnosis was pT_2_N_0_M_0_. The postoperative course was uneventful; no surgical morbidity was evident. Postoperative radiotherapy was not scheduled.

**TABLE 1:**
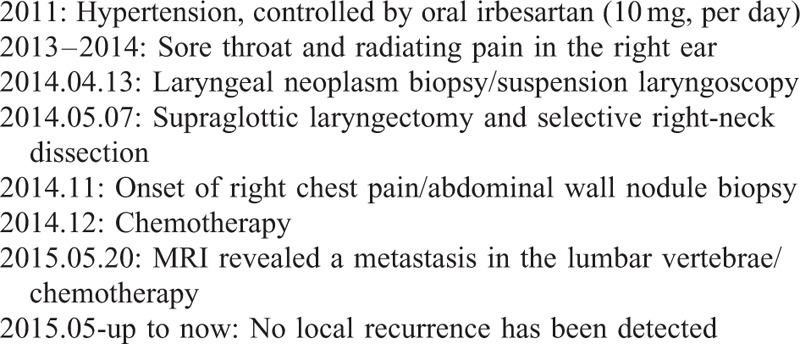
Timeline

### Follow-Up and Outcomes

**FIGURE 5:**
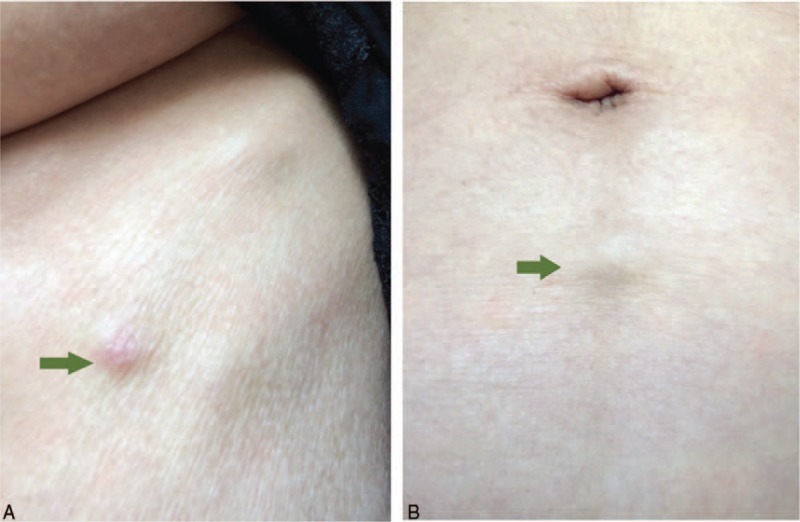
Multiple cutaneous and subcutaneous nodules were evident in the right wall of the chest (A) and the abdomen (B).

The patient received regular monthly follow-ups. On October 9th 2014, PET/CT revealed no recurrence or distant metastasis. In November 2014, the patient complained of pain in the right wall of the chest; multiple cutaneous and subcutaneous nodules were evident at that site and in the abdomen (Figure 5). An abdominal nodule was biopsied and pathology revealed that the atypical metastatic carcinoid had metastasized to both cutaneous and subcutaneous areas of the abdomen. Chemotherapy was then prescribed (capecitabine, 100 mg orally/day and 150 mg orally/night, days 1–14; and temozolomide, 300 mg orally/day, days 2–5). However, even after 2 cycles of chemotherapy, the nodules did not shrink in size and, indeed, several new cutaneous and subcutaneous metastases developed. Pain was not resolved by chemotherapy but was relieved with oral oxycontin. Another chemotherapy regime was then prescribed (octreotide, 30 mg intramuscularly, 1 dose). However, this did not control the pain (scored using a visual analog scale as 6) from the multiple nodules. Pain was relieved with the aid of an intrathecal drug delivery system (ropivacaine, 4 mL + ketamine, 60 mg + morphine, 160 mg; maintenance dose, 6 mg/day). On May 20th 2015, MRI revealed a metastasis in the lumbar vertebrae. Hence, a new chemotherapeutic regime was prescribed (2 cycles: irinotecan, 350 mg/m^2^ by intravenous infusion); the nodules did not respond. Currently, the intrathecal drug delivery system remains in place. No local recurrence has been detected.

**FIGURE 6:**
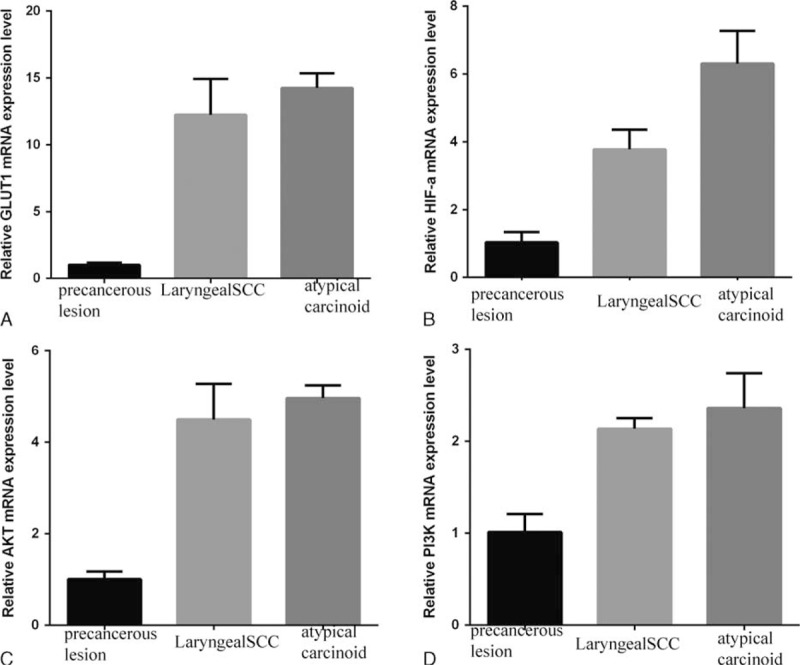
RT-PCR showed that the expression of GLUT-1 (A), HIF-1α (B), p-Akt (C), and PI3K (D) mRNA were high than these in precancerous lesion and laryngeal squamous cell carcinoma. Akt = protein kinase B, GLUT-1 = glucose transporter-1, HIF-1α = hypoxia-inducible factor-1α, PI3K = phosphatidylinositol 3-kinase, RT-PCR = reverse transcription-polymerase chain reaction.

**FIGURE 7:**
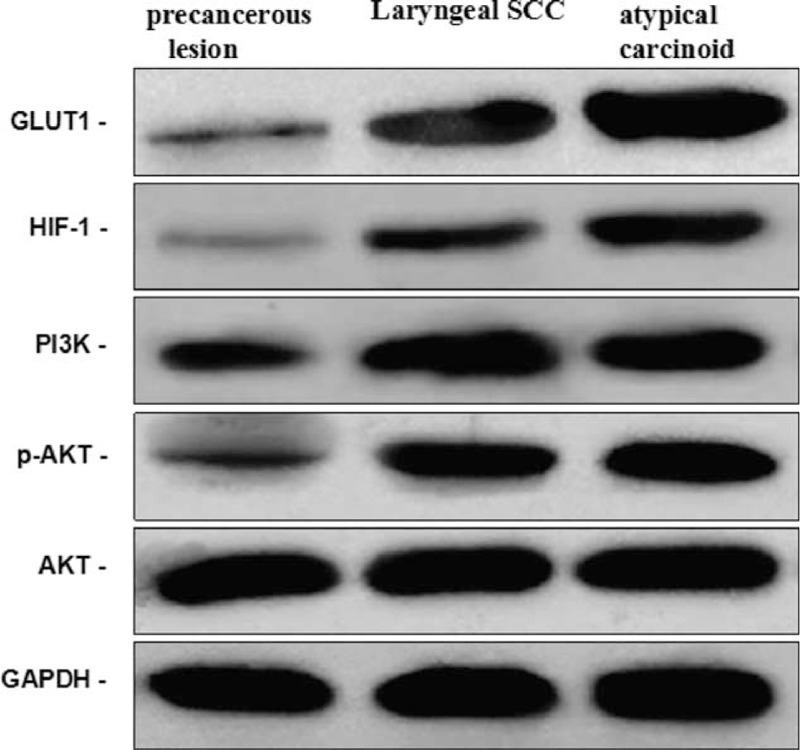
Western blot analysis showed that the expression of GLUT-1, HIF-1α, p-Akt, and PI3K proteins were high than these in precancerous lesion and laryngeal squamous cell carcinoma. Akt = protein kinase B, GLUT-1 = glucose transporter-1, HIF-1α = hypoxia-inducible factor-1α, PI3K = phosphatidylinositol 3-kinase.

GLUT-1, HIF-1α, p-Akt, and PI3K mRNA levels of frozen tissues of primary tumor and precancerous lesion (laryngeal papilloma) as control were measured using real-time reverse transcription-polymerase chain reaction (RT-PCR). The results showed that the expression of GLUT-1, p-Akt, and PI3K mRNA was higher in the atypical carcinoid than in precancerous lesion and in the laryngeal squamous cell carcinoma (Figure 6). GLUT-1, HIF-1α, p-Akt, and PI3K protein levels were analyzed using a BAC Protein Quantification Kit and Western blot analysis. The results showed that the expression of GLUT-1, p-Akt, and PI3K proteins was higher in the atypical carcinoid and in the laryngeal squamous cell carcinoma than in precancerous lesion; however, there is no difference in expression between in the atypical carcinoid and in the laryngeal squamous cell carcinoma (Figure 7). HPV (16, 18, 31, 33, 35, 39, 45, 51, 52, 56, 59, and 68 types), CMV, and EBV DNAs were detected by quantitative fluorescence PCR. During the detection of HPV, H_2_O was used as a negative control and an immunodeficiency virus containing the target gene was the positive control. During the detection of CMV, a CMV-negative sample was the negative control and HCMV AD-169 standard strain was the positive control. During the detection of EBV, saline was the negative control and clinical isolates of the EB virus were the positive control. In all viral detection assays, samples from healthy controls were also included. The results revealed that HPV, CMV, and EBV DNAs were negative. Each primer sample was run in triplicate.

## DISCUSSION

An atypical laryngeal carcinoid is the most common nonsquamous cell carcinoma of the larynx. The tumor is aggressive and the prognosis relatively poor compared to a laryngeal carcinoid. However, the pathogenesis and natural history of, and prognostic factors for, the condition remain poorly understood.

Only 2 reports have explored HPV status in patients with laryngeal NECs.^31,32^ Giordano et al (2009)^31^ found no HPV DNA in a patient with a moderately differentiated laryngeal NEC (an atypical carcinoid). Halmos et al (2013)^32^ described 2 patients who were positive for the high-risk type of HPV (hrHPV) (1 atypical laryngeal carcinoid patient was positive for HPV 18, and 1 patient with a laryngeal small-cell NEC was positive for HPV 16). Both patients experienced relatively good outcomes compared to those of another 5 patients. It was therefore suggested that hrHPV involvement in laryngeal NECs may be associated with good prognoses. We also detected hrHPV in our current patient. We were the first to explore CMV and EBV DNA status in patients with laryngeal NECs. Such patients were negative for hrHPV, CMV, and EBV DNAs. Therefore, any relationship between laryngeal NEC and viral DNA positivity requires further investigations in larger numbers of patients.

Protein p53 is prognostic in terms of the outcomes of patients with neuroendocrine tumors.^33^ Giordano et al (2009)^31^ reported p53 overexpression in a patient with a moderately differentiated laryngeal NEC (an atypical carcinoid). Chung et al (2004)^34^ found that 3 of 6 patients with moderately differentiated laryngeal NECs were p53-positive. McCluggage et al (1997)^35^ also reported that 3 atypical laryngeal carcinoid patients were p53-positive. Overholt et al (1995)^36^ found that 6 of 8 patients with laryngeal neuroendocrine neoplasms (including 2 atypical carcinoids) were p53-positive. The cited authors suggested that p53 mutation may be involved in the pathogenesis of atypical carcinoids.^31,34–36^ Our present patient is also p53-positive.

Hypoxic markers have been explored as possible prognostic factors for NECs, including atypical carcinoids. The markers explored include GLUT-1, HIF-1α, and proteins of the associated phosphatidylinositol 3-kinase/protein kinase B (PI3K/Akt) pathway.^26–28,37–39^ Ozbudak et al (2009)^26^ found that GLUT-1 was expressed in approximately half of all pulmonary NEC patients, and such expression was associated with an increased risk of mortality. GLUT-1 expression is regulated by HIF-1α and the PI3K/Akt signaling pathway.^39^ Hafner et al (2012)^37^ found that the PI3K/AKT pathway was activated in patients with Merkel cell carcinoma and suggested that the pathway may represent a useful new therapeutic target. Kaira et al (2013)^39^ reported high-level immunohistochemical expression of GLUT-1, HIF-1α, and p-Akt in 34 patients with pulmonary NECs. In addition, FDG uptake was associated with the expression of GLUT-1, HIF-1α, VEGF, and CD34. The survival of patients positive for GLUT-1 was poorer than that of others.^39^ In our previous study, we found that GLUT-1, HIF-1α, PI3K, and p-Akt were overexpressed in 10 patients with sinonasal and laryngeal small-cell NECs by 80%, 50%, 40%, and 40%, respectively.^27^ Although the expression levels of GLUT-1, HIF-1α, PI3K, and p-Akt did not correlate with survival in a small patient series, 5 patients who died (100%) were positive for GLUT-1, and 2 (40%) for HIF-1α, PI3K, and p-Akt.^27^ Our present patient expressed high levels of GLUT-1, HIF-1α, PI3K, and p-Akt mRNAs and proteins, as revealed by real-time RT-PCR and Western blot analysis, respectively. Thus, we propose that targeting of hypoxic markers may effectively treat laryngeal NECs.

Although the factors described above may influence pathogenesis and survival, the utilities of such molecular markers require further study. Clinicopathological factors prognostic of laryngeal NEC are well-understood; these include distant metastasis, a tumor diameter over 1 cm and tumor type.^40,41^ Patients with typical laryngeal carcinoids have relatively good prognoses. All atypical laryngeal carcinoids, small-cell NECs and large-cell NECs, are associated with poor outcomes.^40^ Of the latter 3 tumor types, atypical laryngeal carcinoid is the most common form of laryngeal NEC and exhibits a high rate (67%) of distant metastasis.^42,43^ Soga (2003)^43^ analyzed 11,842 cases with carcinoids and variant endocarinomas, and found 199 cases of atypical carcinoids in the larynx with a metastasis rate of 66.8%.^43^ Lymph node metastases were the most common (40%), followed by skin and subcutaneous metastases (20%) and metastases to other sites (40%).^6–8^

Early in 1991, Woodruff and Senie^44^ reported that survival was reduced when tumors triggered cutaneous and subcutaneous metastases. The cited authors reviewed 127 patients with atypical laryngeal carcinoids, 119 (94%) for whom follow-up data were available. Of these 119 patients, 22% developed skin or subcutaneous metastases.^44^ In 2005, Ferlito and Rinaldo^45^ searched the English language literature and found nearly 350 cases of atypical laryngeal carcinoids.^44^ As of 10 years later (in 2015), no further systematic review on this tumor type or metastases thereof has been reported. In the study described herein, we reviewed the English language literature using MEDLINE to run a PubMed/Web of Science search employing the keywords “neuroendocrine carcinoma or atypical carcinoid or moderately differentiated neuroendocrine carcinoma” with “head and neck or laryngeal or larynx” and “extrapulmonary neuroendocrine carcinoma.”

Goldman et al reported the 1st case of atypical laryngeal carcinoid in 1969. The patient also exhibited widespread subcutaneous metastases. Since 2005, an additional 42 cases (including our present case) have been described (Table 2 ).^9,32,41,46–55^ Of these recent cases, 23 were males and 17 females; the genders of 2 were not specified. The male-to-female ratio was thus approximately 1.4:1, lower than those reported by Woodruff and Senie (1991)^44^ and Ferlito et al (2005, 2006).^42,45^ The mean age of the 42 patients was 61 years (range, 38–85 years), and disease incidence peaked in the 5th (12 patients, 28.6%) and 6th (13 patients, 30.9%) decades of life, unlike the data reported by Woodruff and Senie.^44^ Of all cases, 24 (57.1%) had histories of cigarette smoking. Of all tumors, 35 (83.3%) were located in the supraglottic area, 1 (2.4%) in the glottis, 1 (2.4%) in the subglottis, and 4 at undefined laryngeal sites. The main symptoms included sore throat (45.2%), dysphagia (40.5%), and hoarseness (23.8%). Of all patients, 35 (83.3%) received surgery (11 underwent neck dissection), and 10 (28.6%) were prescribed postoperative treatments. Three (7.1%) received radiotherapy alone and 3 (7.1%) received both chemotherapy and radiotherapy. One patient (2.4%) received chemotherapy only.

**TABLE 2:**
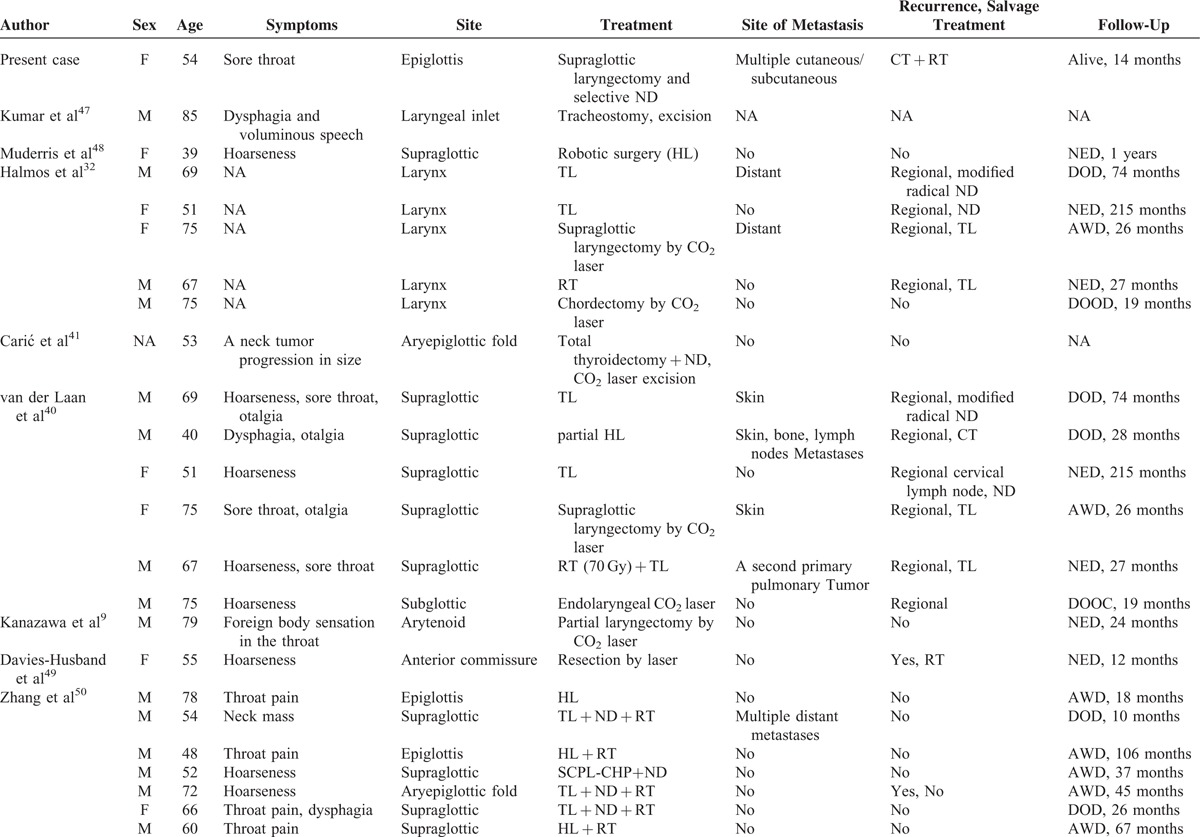
Data on 42 New Cases Described in the English Language Literature Since 2005

**TABLE 2 (Continued):**
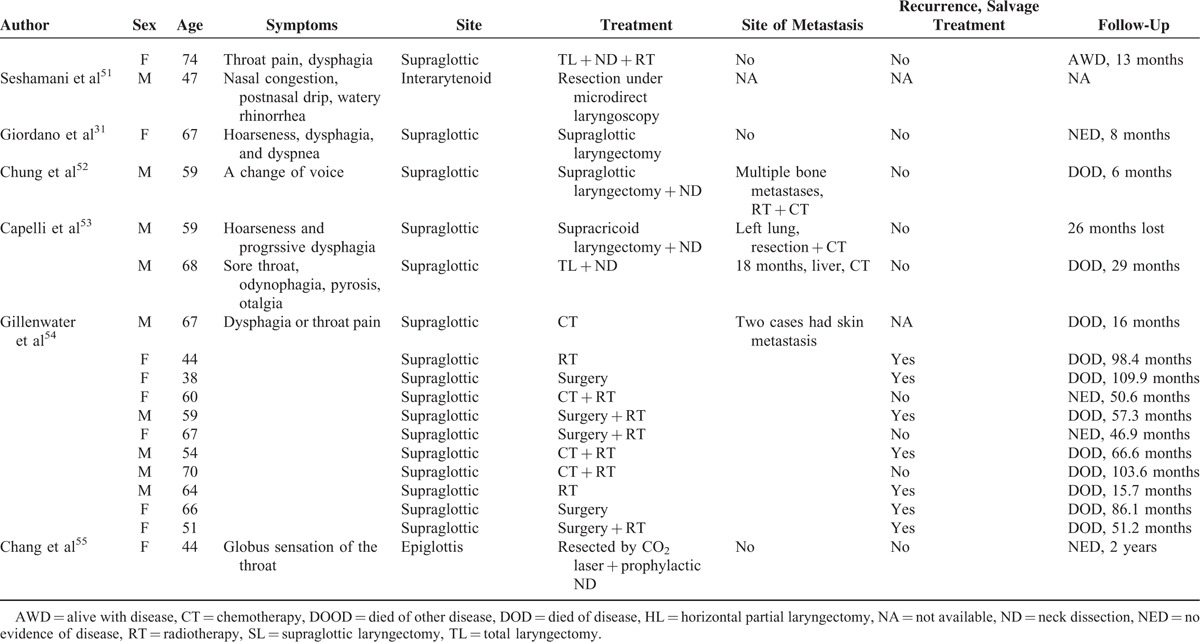
Data on 42 New Cases Described in the English Language Literature Since 2005

The surgical procedures used included total and partial laryngectomy (7 cases were treated using CO_2_ lasers; the others underwent robotic partial laryngectomy^48^). As concluded by Woodruff and Senie (1991)^44^ and Ferlito et al (2005, 2006),^42,45^ surgery remains the mainstream treatment for atypical laryngeal carcinoid today. Recently, transoral robotic partial laryngectomy has become possible.^48^ The principal advantages of robotic partial laryngectomy (compared to the traditional open approaches) are preservation of laryngeal function and elimination of the need for permanent tracheostomy.^48^ However, such surgery is expensive and few hospitals have the sophisticated equipment required. Thus, CO_2_ laser treatment delivered under laryngomicroscopic guidance remains the principal surgical modality for the treatment of early-stage atypical laryngeal carcinoid patients.^9,32,41,49,55^

**FIGURE 8:**
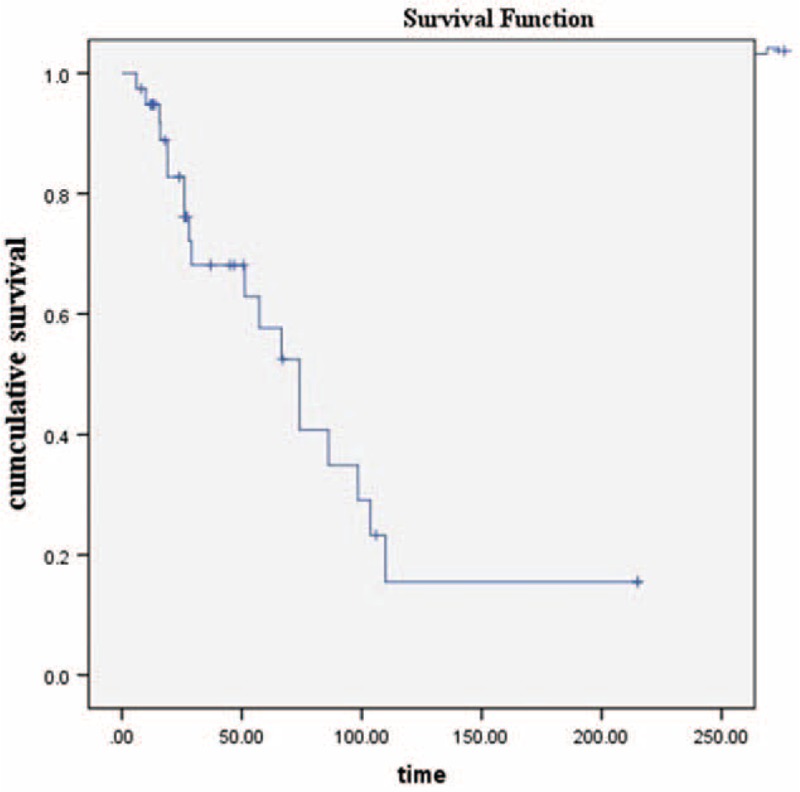
The 5- and 10-year survival rates of 39 patients diagnosed since our last review in 2005 were 57.0% and 15.0%, respectively.

Follow-up information was available for 39 (92.9%) of the 42 patients. Nineteen (48.7%) developed regional recurrences, 12 (30.8%) distant metastases, and 6 (15.4%) both cutaneous and subcutaneous metastases. Overall, 11 patients (28.2%) remained alive and free of disease; 16 41.0%) died from the disease, 2 (5.1%) died from other diseases, 8 (20.5%) were alive with disease, and 1 (2.6%) was lost to follow-up. The 5- and 10-year survival rates were 57.0% and 15.0%, respectively (Figure 8). The 5-year rate was similar to that reported by Woodruff and Senie (1991).^44^ However, the 10-year rate was lower than that reported by the cited authors.^44^ We found that neither the treatment modality used nor the development of local recurrence affected outcomes (both *P* values >0.05). The development of distant metastasis significantly (negatively) affected survival. The estimated mean survival time of patients with distant metastases was poorer than that of others (102.0 vs 41.3 months, *P* < 0.01).

**FIGURE 9:**
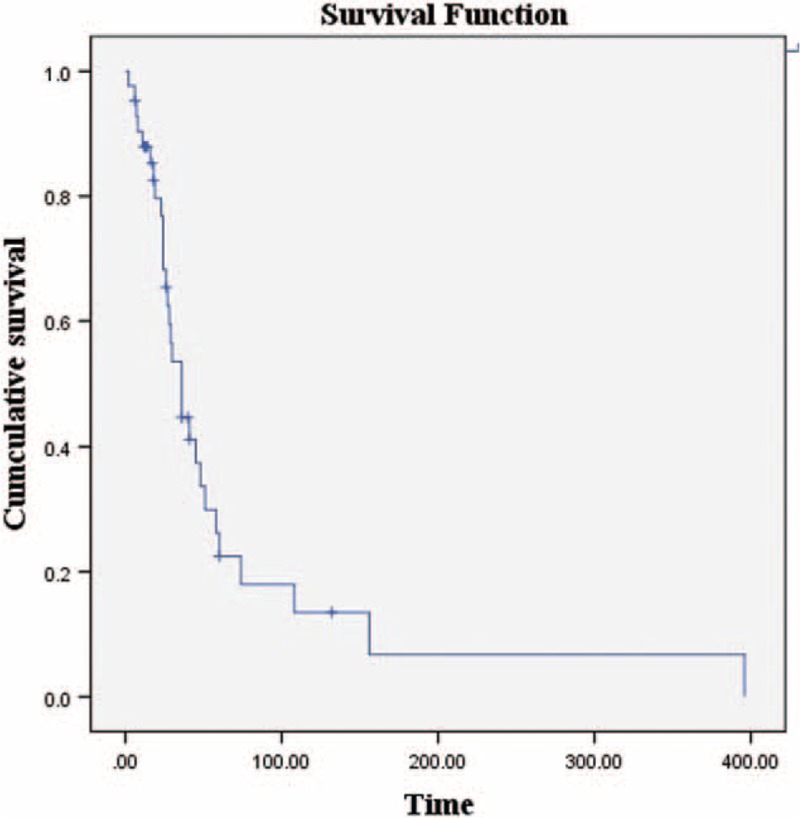
The 3-, 5-, and 10-year survival rates of patients described in the English language literature with cutaneous and subcutaneous metastases from atypical laryngeal carcinoids were 44.0%, 22.0%, and 13.0%, respectively.

We found that the rates of cutaneous and subcutaneous metastasis in these patients were lower (22%) than those reported by Woodruff and Senie (1991).^44^ We searched the English language literature for information on cutaneous and subcutaneous metastases in such patients. We found data on 48 such patients (including our present case) (Table 3 ).^6,8,32,34–36,40,44,54,56,57^ To the best of our knowledge, approximately 392 cases of atypical laryngeal carcinoids have been reported in the English language literature, combining the data of Ferlito et al^42,45^ with those of the present review. The frequency of metastasis in such patients is approximately 12.2%; this incidence may fall as more data accumulate. Thirty patients (62.5%) with such metastases exhibited contemporaneous lymph node invasion. Cutaneous and subcutaneous metastases were always accompanied by distant metastases to other sites, including bone, lung, heart, liver, gallbladder, pancreas, bowel, kidney, ureter, testis, thyroid, diaphragm, and rectus muscle. The principal symptom was pain. Of the 42 patients (87.5%) for whom follow-up data were available, the 3-, 5-, and 10-year survival rates were 44.0%, 22.0%, and 13.0%, respectively (Figure 9).

**TABLE 3:**
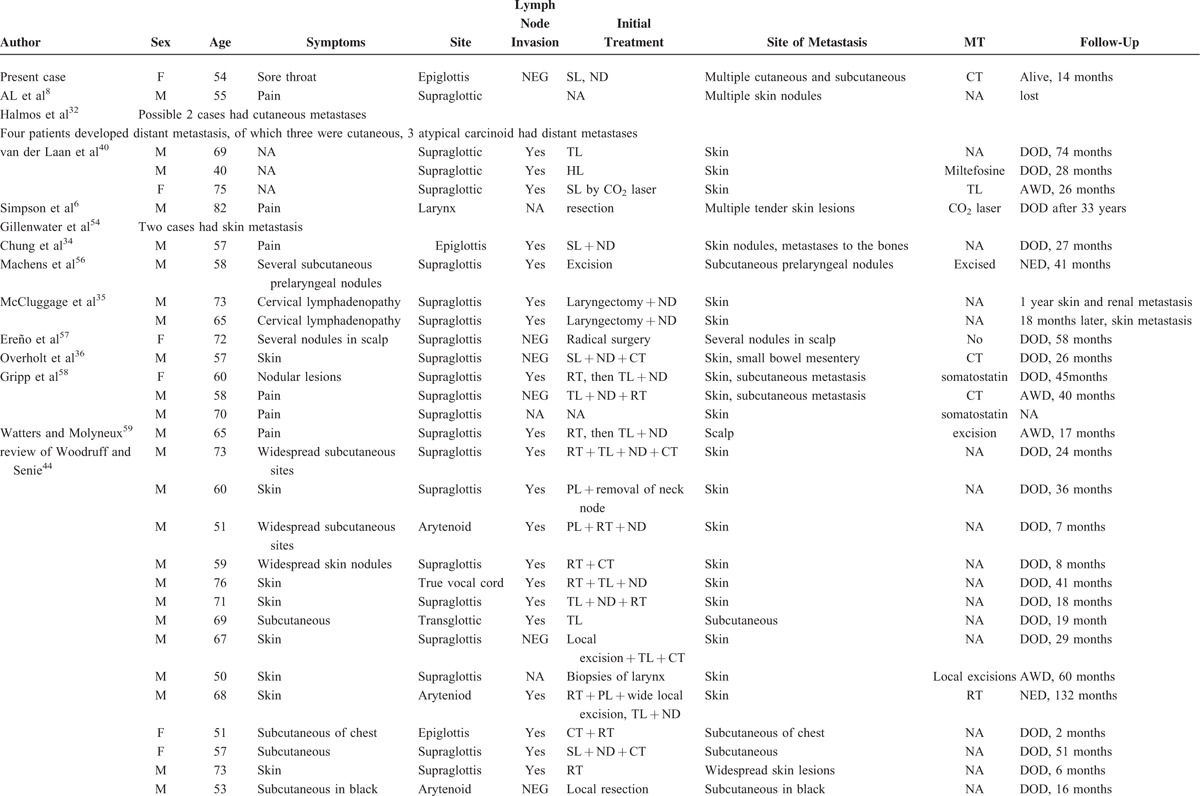
Data on 48 Patients Described in the English Language Literature With Cutaneous and Subcutaneous Metastases From Atypical Laryngeal Carcinoids

**TABLE 3 (Continued):**
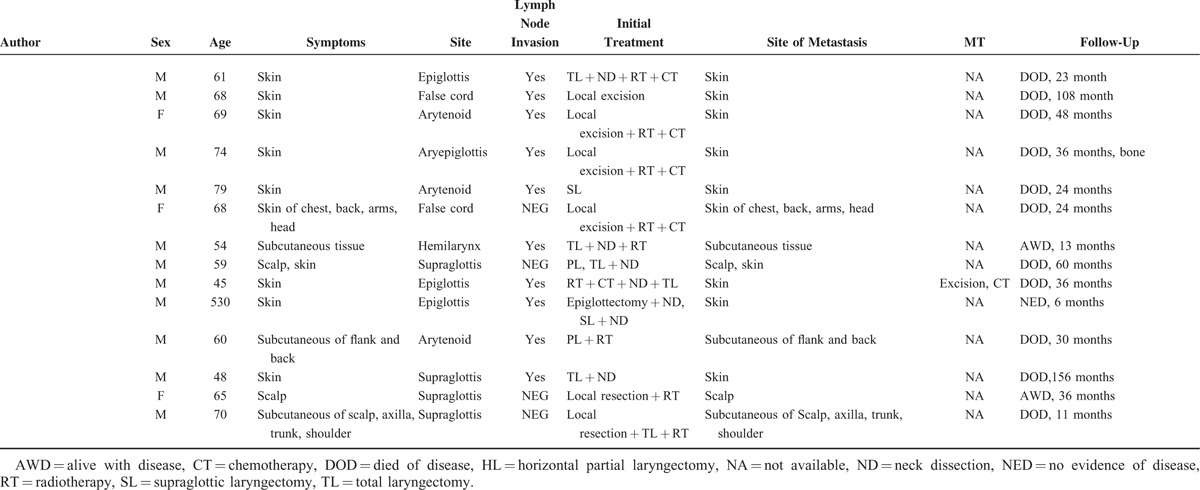
Data on 48 Patients Described in the English Language Literature With Cutaneous and Subcutaneous Metastases From Atypical Laryngeal Carcinoids

No satisfactory treatment for cutaneous and subcutaneous metastases of atypical laryngeal carcinoids has yet been described. Descriptions of treatments are rare.^6,36,40,4,56,58,59^ In 1989, Ferlito and Friedmann proposed that painful skin/subcutaneous metastases should be treated surgically.^44^ van der Laan et al (2012) suggested that salvage surgery, including palliative metastasectomy, had an important role in the treatment of laryngeal NEC. In addition, the overall survival level was reasonable. The cited authors described 3 patients with atypical laryngeal carcinoids who developed cutaneous and subcutaneous metastases; 1 died of disease 74 months after primary treatment (total laryngectomy); 1 received miltefosine after metastasis was diagnosed and died of disease 28 months after primary treatment (horizontal laryngectomy); and 1 underwent total laryngectomy after metastasis was diagnosed and was alive with disease 26 months after primary treatment (supraglottic laryngectomy).^41^ Simpson et al (2009)^7^ reported on an 82-year-old patient who presented with multiple exquisitely tender skin lesions. The patient had undergone resection of an atypical laryngeal carcinoid 33 years prior, and 2 further endoscopic laser resections (with adjuvant radiotherapy) due to local recurrence 2 years prior. The multiple skin metastases were treated using a CO_2_ laser; palliation was effective. However, the patient died from unrelated causes 8 months after the final presentation.^6^ Overholt et al (1995)^36^ described a patient with skin metastases who underwent chemotherapy. The patient died of disease 26 months after primary treatment. It was thus suggested that chemotherapy was ineffective. Gripp et al (1995)^58^ used somatostatin to treat skin metastases from atypical laryngeal carcinoids. However, the drug was no better than other chemotherapies.^58^ Electrochemotherapy is useful to palliate cutaneous and subcutaneous metastases arising from some other malignant tumors.^60^ Such management included intravenous bleomycin (15,000 IU/m^2^); the electrodes were placed under general anesthesia. The overall response rate was 66.6%. Patients with less than 10 nodules and masses smaller than 2 cm in diameter benefited the most.^60^ In our present case, multiple cutaneous and subcutaneous metastases developed 6 months after supraglottic laryngectomy. The tested treatments were unsatisfactory. Pain was relieved only via intrathecal drug delivery.

In conclusion, atypical laryngeal carcinoids are rare, and the prognoses are poor. Prognostic factors include the level of p53, HPV status, certain hypoxic markers, and distant metastasis. The rate of development of cutaneous and subcutaneous metastases was 12.2%. No optimal treatment for such metastases has yet been defined.
